# Network Analysis to Identify MicroRNAs Involved in Alzheimer’s Disease and to Improve Drug Prioritization

**DOI:** 10.3390/biomedicines14010147

**Published:** 2026-01-11

**Authors:** Aldo Reyna, Simona Panni

**Affiliations:** Dipartimento di Biologia Ecologia Scienze Della Terra (DiBEST), Università Della Calabria, Via Pietro Bucci Cubo 6C, 87036 Rende, CS, Italy

**Keywords:** microRNA, mimics, biocuration, RNA–protein interactions, multi-omics data analysis

## Abstract

**Background**: Advances in the understanding of molecular mechanisms of human diseases, along with the generation of large amounts of molecular datasets, have highlighted the variability between patients and the need to tailor therapies to individual characteristics. In particular, RNA-based therapies hold strong promise for new drug development, as they can be easily designed to target specific molecules. Gene and protein functions, however, operate within a highly interconnected network, and inhibiting a single function or repressing a single gene may lead to unexpected secondary effects. In this study, we focused on genes associated with Alzheimer’s disease, a progressive neurodegenerative disorder characterized by complex pathological processes leading to cognitive decline and dementia. Its hallmark features include the accumulation of extracellular amyloid-β plaques and intracellular neurofibrillary tangles composed of hyperphosphorylated tau. **Methods**: We built a protein interaction network subgraph seeded on five Alzheimer’s-associated genes, including tau and amyloid-β precursor, and integrated it with microRNAs in order to select regulated nodes, study the effects of their depletion on signaling pathways, and prioritize targets for microRNA-based therapeutic approaches. **Results**: We identified nine protein nodes as potential candidates (Pik3R1, Bace1, Traf6, Gsk3b, Akt1, Cdk2, Adam10, Mapk3 and Apoe) and performed in silico node depletion to simulate the effects of microRNA regulation. **Conclusions**: Despite intrinsic limitations of the approach, such as the incompleteness of the available information or possible false associations, the present work shows clear potential for drug design and target prioritization and underscores the need for reliable and comprehensive maps of interactions and pathways.

## 1. Introduction

Over the past two decades, the generation of an unprecedented quantity of molecular data has profoundly transformed both research and clinical practice, significantly advancing precision medicine. The increased understanding of disease molecular mechanisms has made it possible, particularly in oncology, to identify specific therapies that target mutated genes or non-functional proteins and to stratify patients who are most likely to benefit from them [1]. Imatinib, Osimertinib and Vemurafenib are examples of kinase inhibitors that have proven effective in cancer patients carrying mutations in ABL, EGFR and BRAF, respectively [2].

Similarly, antisense oligonucleotide-based therapies have been developed and, in some cases, approved by the FDA, such as Nusinersen against spinal muscular atrophy or Eteplirsen for Duchenne muscular dystrophy [3,4]. In this context, therapeutic targeting of non-coding RNAs (ncRNAs) has generated great interest, particularly in microRNAs, which are relatively easy to target and among the best characterized. While the strongest gene regulation occurs at the transcriptional level, microRNAs modulate gene expression by acting on messenger RNAs, and numerous studies have confirmed that their dysregulation is a hallmark of several diseases. The mature “guide” strand of miRNA recognizes its targets by binding to short complementary regions, typically located in the 3′ untranslated region of the mRNA [5]. The interaction very often results in mRNA degradation through deadenylation, decapping and decay, as observed by qRT-PCR in most published studies, whereas in other cases the mRNA level remains unchanged, but protein production decreases due to translational repression [6].

MicroRNAs have emerged as promising therapeutic targets in numerous pathologies, including Alzheimer’s disease (AD), the predominant form of dementia, which affects more than 55 million people worldwide and for which traditional medication has very limited efficacy [7]. AD can be categorized as early-onset if it manifests before the age of 65 or as late-onset if it manifests later. Patients with AD exhibit substantial accumulation of amyloid-β plaques (Aβ) and neurofibrillary tangles (NFTs) within their brains. These proteinopathies are accompanied by chronic neuroinflammation, microglial and astrocytic activation and oxidative stress, which collectively contribute to widespread neuronal loss and neuronal disconnection [8].

It has been reported that miR29a and miR107 are downregulated in AD brains and that their levels inversely correlate to (Aβ) plaques and neurofibrillary tangles [9,10]. Conversely, miR34c upregulation correlates with impaired memory functions in mouse models [11]. Other miRNAs, such as miR-146a and miR-155, are involved in microglia-mediated inflammatory responses, and their modulation has been reported to attenuate neuroinflammation and neuronal damage [12].

Recent reviews report an increasing number of microRNAs potentially involved in AD [13,14,15], encouraging the development of miRNA-based medications.

Two major classes of molecules based on microRNAs have been developed to down- or up-regulate specific genes: microRNA mimics and antimiRs [16].

In brief, mimics are synthetic double-stranded RNA molecules that match the corresponding miRNA sequences, therefore replacing their functions and downregulating specific genes. AntimiRs are single-stranded RNAs designed to target and inhibit microRNAs, thereby increasing expression of the target genes. The effects of both drugs entirely rely on the roles of the genes that the microRNAs affect [16,17]. It should be considered, however, that any phenotype reflects the interplay of multiple molecular components, connected in complexes and pathways, and that, in cells, the information flows through dynamic networks of molecular interactions, where the nodes are bio-molecules, including proteins, and edges represent functional associations, including physical protein–protein interactions [18]. In the present paper we introduce the use of network analysis to model the effect of mimics or antimiR, which may be useful for Alzheimer’s disease (AD) treatment, on protein communication and network robustness [19]. Our work is grounded in the disease module concept, which posits that genes and proteins implicated in a given pathology tend to cluster within specific, topologically coherent regions of the interactome [18,19]. Disease-associated genes were shown to form connected subnetworks (“modules”), whose internal cohesion and separation from the rest of the interactome determine how perturbations propagate and how diseases relate to each other [18,20]. Later work in network medicine has reinforced and expanded this view, demonstrating that disease modules are often characterized by high local connectivity, enrichment of central or bridging nodes and vulnerability to targeted structural disturbance [21]. Accordingly, the analysis of the network topology is a powerful way to assess the susceptibility to targeted node downregulation, and removing individual proteins from a disease module can reveal their contribution to module integrity in terms of loss of connectivity, fragmentation of local neighborhoods or increased network distance among disease-gene members, which is particularly relevant for pharmacological approaches aiming at identifying key factors for the development of new drugs.

Over the last year, the IMEx Consortium’s team of expert curators [22,23] have undertaken the effort to annotate experimental interactions involved in Alzheimer’s disease, building a disease-related interactome to support research in neurodegenerative diseases and to study variant-specific perturbations [24]. Accurate and standardized biocuration is essential for producing reliable network-based analyses, particularly in protein–protein interaction studies. The IMEx Consortium provides one of the most rigorously curated, experimentally validated PPI resources available. Each interaction is manually reviewed, annotated with controlled vocabularies, linked to primary experimental evidence, and assigned confidence metrics, ensuring both transparency and reproducibility. Such curation is critical for avoiding artifacts that may arise from heterogeneous data sources or automated text-mining errors.

In the present work, we computed the centrality measures of a group of proteins involved in the Alzheimer’s disease module and targeted by microRNAs, and we compared the effect of node depletion in the context of protein networks collected by the consortium and composed of high-confidence binary associations [24,25]. The results both demonstrate how the analysis of network vulnerability to specific node deletion facilitates target prioritization and mimics-based therapeutic approaches and highlight the urgency of completing the biocuration of high-quality interaction networks integrated with ncRNAs regulation.

## 2. Materials and Methods

### 2.1. Construction of an Integrated microRNA–Protein Disease Module

A set of five proteins well known to be involved in AD was selected. These included the amyloid precursor protein (App, UniProt ID: P05067), microtubule-associated protein tau (Mapt, P10636), presenilin-1 (Psen1, P49768), presenilin-2 (Psen2, P49810) and beta-site App cleaving enzyme 1 (Bace1, P56817). Protein–protein interactions for each protein were retrieved independently from three databases: STRING [26], BioGRID [27] and IntAct [25]. The analysis was restricted to the human species (*Homo sapiens*, taxonomy ID: 9606). To retain only the most reliable experimental interactions, a specific set of parameters was introduced to filter the interaction data appropriately. Data were retrieved from STRING via the STRING API (accessed on 10 July 2025), with the physical interactions setting selected and a minimum confidence score of 0.9. For BioGRID, interactions were obtained using the BioGRID REST API (accessed on 10 July 2025), filtering for physical interactions supported by low-throughput experimental techniques. The database does not provide a built-in confidence score, so we evaluated the strength of each interaction using the number of supporting papers and applying a cut-off of two papers. IntAct was accessed via the Cytoscape App 3.10.3 [28] on 11 July 2025, filtering for “direct interactions” and “physical associations”. A minimum confidence score (MI score) of 0.60 was applied [29].

To integrate the microRNA regulative network into the protein–protein interaction web, strong interactions supported by luciferase reporter assays were retrieved from the miRTarBase [30] and from IntAct (both accessed on 10 August 2025) and filtered as described elsewhere [31]. The dataset was processed using a custom Python 3.13 script to retain only human microRNAs that bind genes included in the previously assembled PPI network and to filter it based on the number of papers supporting the interaction using the luciferase assay method.

### 2.2. Literature Mining to Search Alzheimer-Related microRNAs

We conducted a systematic literature search to identify microRNAs associated with Alzheimer’s disease in brain tissues and compare them with those integrated in the network. Two biomedical literature databases were accessed programmatically using Python: Europe PubMed Central (Europe PMC) and PubMed (via NCBI Entrez). The RESTful API of Europe PubMed Central was used, and the search was refined to exclude review articles and restricted the results to human studies. A parallel literature search was performed in PubMed using the Bio.Entrez module of the NCBI Entrez Programming Utilities (E-utilities). The query string was adapted to match PubMed’s syntax and focused on the title and abstract fields. The datasets obtained were merged and deduplicated according to PMID identifiers, and the resulting dataset was manually reviewed to obtain a set of 53 articles reporting 66 microRNAs that regulate genes involved in AD.

### 2.3. Statistical Analyses

To evaluate the enrichment of the network in disease-associated genes, we retrieved a list of genes from Open Targets [32] (accessed on 5 August 2025), filtered them for a global score > 0.38, which corresponds to a high to medium confidence value (i.e., associations supported by multiple sources of evidence), and thus identified 300 of the 9500 genes listed as associated with AD. Then we performed a comparative analysis to identify the overlapping elements. Fisher’s exact test was applied, with 19.435 protein-coding genes (GENCODE v47) as the reference background. For microRNAs, the list of AD-related microRNAs was extracted from the literature as described above, and the reference background was estimated considering only the mature miRNAs that were supported by strong evidence [33].

Molecular function enrichment was performed using ClueGO in Cytoscape [34], setting “All_Exp” evidence and hypergeometric test *p* ≤ 0.05.

### 2.4. Protein and microRNA Expression in Brain Tissues

**Figure 1:**
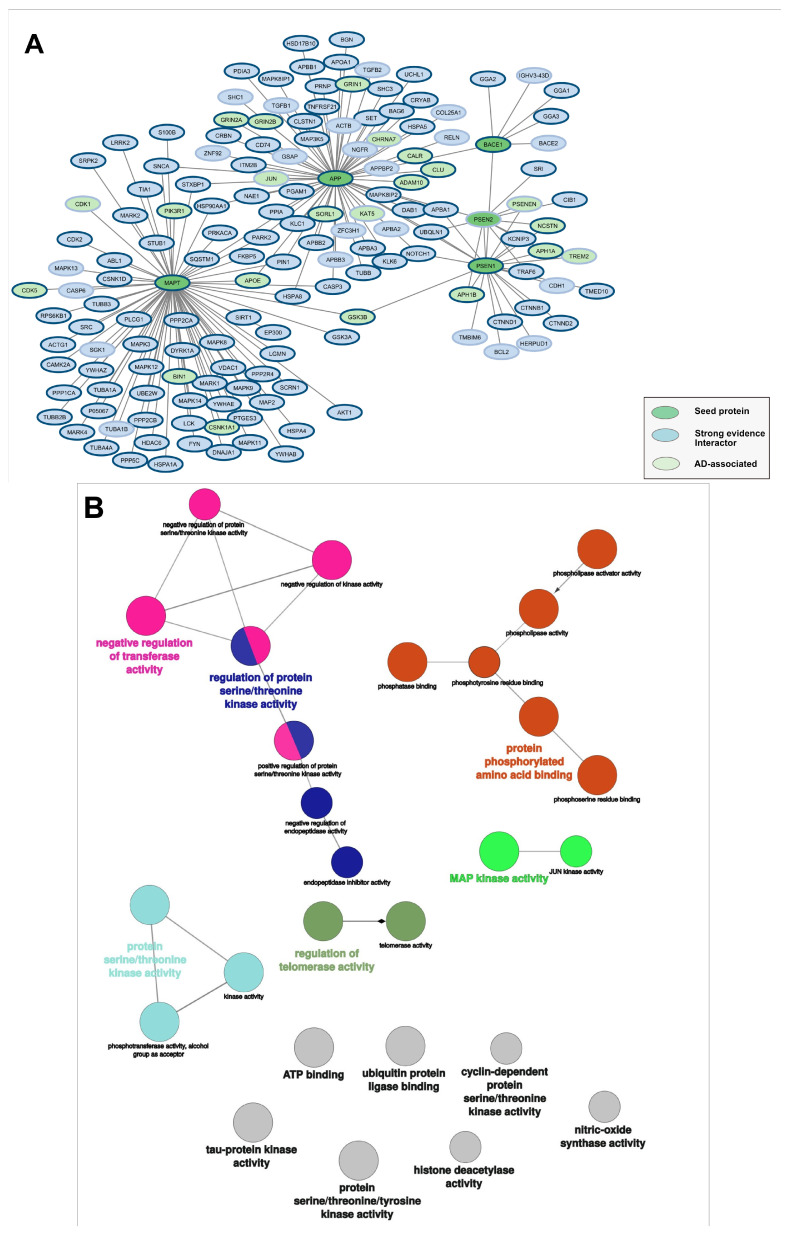
Alzheimer’s disease module. (**A**) The graph was seeded on 5 genes involved in AD: APP, MAPT, BACE1, PSEN1 and PSEN2. The network is highly enriched in other disease-associated genes: pale green nodes are Open Targets AD-associated genes. Blue borders of the nodes indicate genes expressed in the frontal cortex in Alzheimer’s patients according to [35]. (**B**) Network visualization of significantly enriched GO molecular function terms (Benjamini–Hochberg adjusted *p* ≤ 0.05) in first neighbor interactors of MAPT, generated by ClueGO in Cytoscape [34], showing a strong enrichment of kinase-related functions.

The protein abundance of each node in the frontal cortex of Alzheimer’s disease was evaluated from [35], and all proteins detected with more than 1 peptide were considered expressed in Figure 1.

The expression level of microRNAs was downloaded from miRNATissueAtlas 2025 [36] (accessed on October 2025), and the values of the brain hippocampus and brain cortex were normalized and used for the heatmaps.

### 2.5. Other Networks Construction and Centrality Measures

Human Protein Network: Protein interaction data were obtained from the IntAct database, accessed on 11 September 2025, in miTAB 2.5 and filtered for *Homo sapiens* (NCBI Taxonomy ID 9606) and an IntAct MI score of 0.65 or higher [29].

Alzheimer’s Disease Network: The dataset of interactions occurring among proteins involved in AD [24] was downloaded from the IntAct portal (https://www.ebi.ac.uk/intact/download/datasets) accessed on 11 September 2025, in miTAB 2.5 and filtered for *Homo sapiens* and an IntAct MI score of 0.65 or higher.

Both networks were constructed using the igraph package in R.

Only the source and target protein identifiers were extracted to build an undirected graph in which nodes represent proteins and edges represent experimentally supported interactions. Standard network centrality measures were computed for selected nodes using the igraph package in R to assess the topological relevance of individual proteins. Specifically, Degree Centrality was calculated as the number of edges of the node. Betweenness centrality was calculated as follows:

$$Cb(v)=\sum_{s\neq v\neq t}\frac{\sigma st(v)}{\sigma st}$$

where *σst* = total number of shortest paths between nodes *s* and *t* and *σst*(*v*) = number of those shortest paths that pass through node *v*.

Closeness Centrality was calculated as follow:

$$Cc\left(v\right)=n-1/\sum_{u\neq v}d(v,u)$$

where *n* is the total number of nodes in the connected component containing *v* and *d*(*v*,*u*) is the shortest path distance between node *v* and node *u*.

These metrics were computed across the human interactome and the AD interactome.

### 2.6. Node Depletion Analyses

For each node of interest, an in silico node depletion experiment was conducted.

Each node was individually removed from the network, and the resulting topology was analyzed in terms of the number of connected components, the size of the largest connected component, and changes in the average shortest path length, using the igraph package in R.

### 2.7. Local Networks Visualization

Local node-centric networks were obtained using the Pandas and Python igraph libraries. The networks were extracted for six proteins of interest: P31749 (Akt1), P49841 (Gsk3b), P56817 (Bace1), Q16644 (Mapk3), P27986 (Pik3r1) and P02649 (Apoe). These local networks were defined as all vertices within a geodesic distance of two or less from each seed node. For interpretability, nodes were classified by ring level: seed (ring 0), first-degree neighbors (ring 1) and second-degree neighbors (ring 2). Each hub-centric network was exported to GraphML format and visualized in Gephi 0.10 using community-aware and force-directed layout algorithms.

## 3. Results

### 3.1. Alzheimer-Associated Genes Interact Preferentially with Other AD-Linked Genes

A disease is rarely the consequence of a mutation in a single gene, but rather reflects the interplay of multiple disease-associated components, which have a propensity to interact with each other, forming connected subgraphs that have been defined as disease modules [18].

To assemble a “disease module” related to Alzheimer’s disease, we built a subgraph seeded on three proteins coded by genes strongly associated with AD: amyloid precursor protein (APP), presenilin1 (PSEN1) and presenilin 2 (PSEN2). Familial AD is characterized by autosomal dominant genetic mutations in these genes, and these mutated genes also appear in the sporadic form of the disease [7]. Moreover, both familial and sporadic forms of AD are characterized by the accumulation of amyloid beta peptides, which originate from App through the proteolytic activity of presenilin 1 and 2. We also considered Bace1, another enzyme involved in app proteolytic cleavage. The fifth seed protein is tau (MAPT), the major component of the neurofibrillary tangles, which strongly correlates with clinical symptoms.

The interactors of these five proteins were retrieved by combining data from three sources—IntAct [25], STRING [26] and BioGRID [27]—and filtering for strong evidence to minimize false positives and background noise. After removing duplicates, the resulting subgraph was composed of 156 nodes and 179 edges, as illustrated in Table 1 and Figure 1.

Almost 80% of the proteins included in the subgraph have been detected in quantitative proteomic studies on the frontal cortex of Alzheimer’s patients [35] (nodes with a blue border in Figure 1), confirming their expression during the disease.

**Figure 2:**
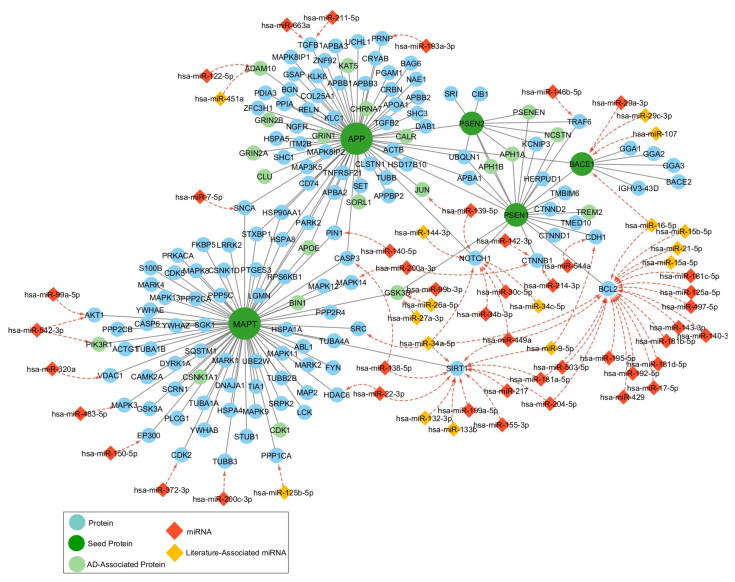
AD-disease module integrated with microRNAs regulatory interactions. Red and yellow diamonds indicate miRNAs directly binding to the mRNA of the proteins; the yellow miRNAs have also been reported in the literature as potentially involved in Alzheimer’s disease.

We compared the molecular components of the first neighborhood of the five selected seed nodes with the genes listed in Open Targets as associated with the disease (association score filtered at 0.38). As expected, we found that the subnetwork was significantly enriched in other Alzheimer-associated genes (Table 2, Fisher test *p*-value < 2.2 × 10^−16^). The proteins corresponding to AD-associated genes are highlighted in light green in Figure 1A and in Figure 2.

Since the accumulation of aberrantly hyperphosphorylated tau proteins is a hallmark of the disease [37], we analyzed the functional enrichment of Mapt interactors, which remarkably confirms a kinase-driven regulation for this protein, showing a significant enrichment of phosphorylation-related molecular functions (Figure 1B).

### 3.2. Prediction of microRNAs Potentially Involved in the Disease

Given that the main function of microRNAs is to regulate the expression level of genes, the molecular processes and the diseases in which they are involved largely, if not completely, depend on the targets [5]. To identify microRNAs related to AD, we integrated the disease module described above with microRNA associations. To this aim, in order to filter indirect and faint interactions, we considered only microRNAs whose binding with one of the nodes was supported by “luciferase assay” in at least two different experiments [38,39].

As shown in Figure 2, approximately 20% of the 156 nodes are regulated by microRNAs, and only three nodes—Sirt1, Bcl2 and Notch1—are regulated by many, in accordance with what has been observed elsewhere [31].

Table 3 lists the miRNAs that regulate the AD module. Among them, miR-29a-3p, miR-29c-3p and miR-107, regulate beta secretase Bace1, responsible for the proteolytic cleavage of the amyloid precursor protein in a soluble extracellular domain, and a carboxyl-terminus fragment named C99. This fragment is further processed by the gamma-secretase complex (comprising the above-mentioned Psen1 and Psen2), producing amyloidogenic peptides that accumulate in nervous tissue during the disease. The overexpression of miR-29a-3p, miR-29c-3p or miR-107 may therefore diminish the production of toxic peptides.

Three microRNAs—miR-99b-3p, miR26a-5p and miR27a-3p—downregulate Gsk3b, which in turn phosphorylates Mapt on several residues, promoting the formation of the disease’s neurofibrillary tangles. Other kinases, which interact with Mapt, are regulated by microRNAs, and among them, Cdk2 is regulated by miR372-3p, Mapk3 by miR483-5p and Akt1 by miR542-3p. MiR-146b-5p regulates Traf6, which mediates Toll-like receptor (TLR) and Interleukin 1 receptor (IL-1R) signaling and subsequent activation of NF-kappaB [40]. This cascade is part of the brain’s innate immune system, activated by amyloid beta accumulation, and leads to the expression of pro-inflammatory cytokines. The reduction in Traf6 may have beneficial effects on amyloid-induced inflammatory cell death.

Two microRNAs bind to Adam10 mRNA: miR-122-5p and miR-451a. Adam10 is a transmembrane protease that cleaves the extracellular domains of membrane-anchored proteins in a process termed ectodomain shedding. This cleavage occurs both in the proteolysis of Notch receptors and in the non-amyloidogenic α-secretase cleavage of App [41].

Adam10, therefore, contrasts the progression of the pathology, reducing amyloid beta generation [33,42], and miR-122-5p or miR-451a overexpression may have the opposite effect.

We compared the list of microRNAs with an independently curated set of Alzheimer-related microRNAs assembled via text-mining and manual review of the literature. Seventeen miRNAs were found in both sets (the yellow hexagons in Figure 2), indicating that the microRNAs listed are significantly enriched in AD-related genes (Exact Fisher test *p*-value = 3.19 × 10^−9^).

**Figure 3:**
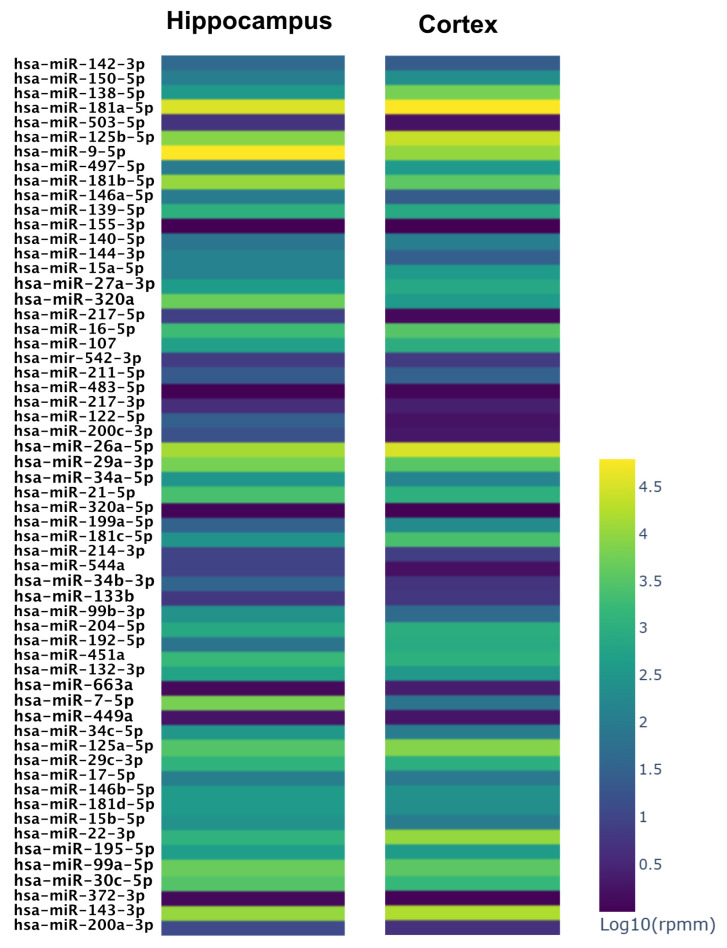
MicroRNA expression levels in two brain tissues [21]. The values have been normalized in reads per million mapped reads (RPMM). Yellow lanes represent very high levels of expression (Supplementary Table S1).

We evaluated the expression levels of the microRNAs listed in Table 3 in brain tissues most impaired during neurodegenerative diseases: the hippocampus and frontal cortex. As shown in Figure 3 and Supplementary Table S1, the microRNAs acting on Cdk2, Mapk3 and Akt1 show a poor expression level, as miR122-5p acting on Adam10, while most of the other miRNAs acting on the disease module have a moderate to high expression level in normal brain hippocampus and cortex tissues (Supplementary Table S1, Figure 3).

### 3.3. Centrality Measures and In Silico Knock-Out Experiments in the Human Interactome

Once we pinpointed those nodes that could be targeted with synthetic microRNAs to alter the pathology development, our goal was to determine whether these nodes were indispensable, neutral or dispensable for the vulnerability of the physiological and pathological ensemble of interactions [43]. To this end, we mapped the nodes in the context of the whole human interactome and investigated how sensitive these networks are to the removal of each node.

The human interactome was downloaded from IntAct and filtered for high-confidence binary interactions with an MI score greater than 0.65 [29]. The analyzed network was composed of 8865 nodes and 124,343 edges. Standard network centrality metrics were computed to characterize the topological role of the selected nodes. In particular, as shown in Table 4, the betweenness centrality was computed to assess the extent to which a node lies on the shortest paths between other proteins and its role in maintaining the information flow. Closeness centrality was used to estimate how efficiently a protein can reach other nodes in the network and how quickly it can influence them. While node degree is a purely local centrality measure, which considers the number of interactions of each node, betweenness and closeness represent global centrality measures that account for the shortest paths among all pairs of nodes and help to analyze the heterogeneous structure of complex networks [20]

**Figure 4:**
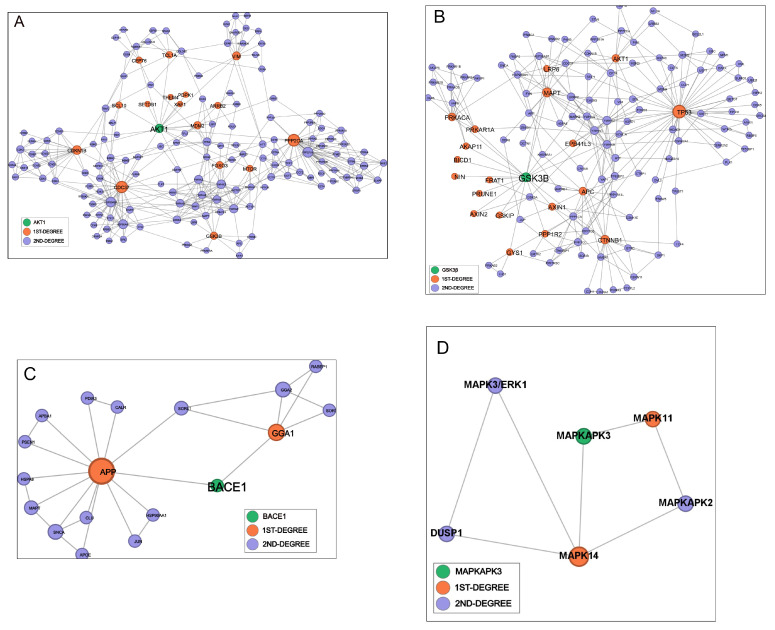
The local networks of Akt1 (**A**), GSK3β (**B**) Bace1 (**C**) and Mapk3 (**D**) showing first- and second-degree interactors extracted from the human IntAct interactome (MI score ≥ 0.65). Nodes are colored by distance from the seed (green: seed; orange: 1st neighbors; purple: 2nd neighbors) and sized by degree within the subgraph. Edges denote physical PPIs; layout: ForceAtlas2 (Gephi).

According to the computed parameters (Table 4), Traf6, Gsk3b and Akt1 are bottlenecks and pathway connectors showing high degrees and high betweenness (Figure 4A,B), and their removal increases network fragmentation and decreases communication efficiency. Cdk2, Pik3R1, Adam10 and Apoe are dense local hubs but do not bridge big modules, having a moderate degree and betweenness, and behave as minor connectors (Supplementary Figure S1). Mapk3 and Bace1 map on peripheral branches, so their loss is largely irrelevant for network structure (Figure 4C,D).

To further assess the functional impact of these nodes on global network structure, we performed in silico experiments, sequentially depleting each node from the network to assess the possible formation of disconnected components. Each of the selected nodes was individually removed from the network, and the resulting topology was analyzed in terms of the number of connected components, the size of the largest connected component and changes in the average shortest path length. This approach simulates the potential disruptive effect of targeting key proteins, providing insight into the network’s robustness and vulnerability [20,43,44].

The minimal variation in the number of components indicates that the deletions cause only minor fragmentation and suggests that the global network structure is quite stable, with one to four additional components created by the lost connections (Table 5). Accordingly, the largest component size (LCC), which measures the number of nodes remaining in the giant connected core after the deletions, shows little fluctuation. The largest increase in average path length, which indicates communication efficiency, was observed after removing Traf6; however, Gsk3b and Akt1 also caused fragmentation. Adam10’s and Apoe’s roles as moderate local connectors were confirmed, while Mapk3 and Bace1 had practically no impact, indicating they are not central connectors and can be downregulated with limited effects on the other pathways.

### 3.4. In Silico Knock-Out Experiments in Alzheimer-Focused Interactome

The IMEx Consortium has recently assembled a comprehensive collection of protein interactions investigated in the context of Alzheimer’s disease or affected by clinical variants [24]. To evaluate the structural contributions of the previously analyzed nodes to this focused network, we computed centrality metrics and measured its topological robustness. To facilitate comparison with previous experiments, the AD network was filtered, retaining only interactions with an MI score > 0.65. The resulting network was composed of 771 nodes and 3590 edges.

In this smaller AD-focused interactome, among the tested nodes, Adam10 emerged as the most critical: it is the highest-degree protein, and its removal causes the largest topological disruption, increasing the number of components by four and reducing the large, connected component by nine nodes (Table 6 and Table 7). Its deletion also markedly reduces the average path length, consistent with the loss of peripheral branches and the consequent contraction of the network core. Deletion of Apoe results in a moderate but clearly detectable perturbation of network topology: it increases the number of connected components by one and reduces the size of the LCC by four nodes, indicating that it contributes to maintaining the connection of a small submodule to the main network core. The marked decrease in average path length suggests that it lies in an elongated branch, and its removal trims distal regions, yielding a more compact remaining core. Gsk3b also impacted the network, a role consistent with a connector hub bridging small subnetworks, increasing fragmentation of one component and decreasing the LCC by two nodes, in line with its moderate centrality values. Bace1 deletion had only minimal effects, which confirms that it resides on a peripheral branch; its removal causes the detachment of one node only and no variation in the number of components. Traf6 and PIK3R1 removal exhibited no detectable effect, consistent with a low-centrality position in the AD interactome.

To gain more insights into this last observation, and to evaluate the effect of three nodes not included in this small collection of interactions, we repeated the analysis on the AD network [24] filtered with a lower score (MI score > 0.56) to include more interactions, slightly increasing the noise (Supplementary Tables S2 and S3).

Adam10 and Gsk3b were confirmed as possible bottlenecks, and their removal impairs global communication. Bace1 and Traf6 were confirmed to be peripheral nodes, biologically important but largely irrelevant for the network structure. Surprisingly, PIK3R1, which was linked to one other node in the small AD network in this less stringent collection of interactions, becomes a highly connected hub, bridging other components, clearly demonstrating the importance of choosing the appropriate set of interactions to conduct meaningful analyses (Table 6 and Table S2).

## 4. Discussion

Mutations and variations in gene expression from person to person result in very different healthy or pathological phenotypes, according to their genotype and to other variables.

The observed variability is consistent with the modern understanding of “disease modules”, which conceptualizes complex disorders as perturbations of tightly interconnected subnetworks rather than isolated gene defects. The present paper highlights the relevance of comprehensive and systematic characterization of these subnetworks to gain a full understanding of the molecular basis of the diseases and of how individual components of the network contribute to the phenotype in physiological and pathological conditions. Node depletion analysis applied to microRNA target nodes provides a powerful systems-level framework to support mimic-based drug prioritization. By computationally simulating the downregulation of microRNA targets within the human interaction network and a disease-specific network, this approach captures the network-wide consequences of microRNA mimic activity rather than focusing on single-gene effects.

In this framework, proteins such as Gsk3b, whose deletion fragments the human interactome, likely occupy essential positions at the boundaries of functional modules (Figure 4B). Their removal led to significant increases in path length, and as a result, they can be prioritized as structurally or functionally central in the Alzheimer’s-related interactome. Acting on these nodes, however, could trigger effects beyond the intended target, reaching pathways unrelated to the pathology. Biological networks are highly tolerant to random failures but become extremely vulnerable when key central nodes are disrupted [19]. On the other hand, proteins lying on peripheral branches, such as Bace1 (Figure 4C), may have a limited but more focused effect. The use of mimics simulating miR-29a-3p, miR-29c-3p or miR-107 acting on Bace1 (Figure 2 and Table 3) may shift the balance between the toxic, amyloidogenic pathway and the non-amyloidogenic App cleavage by alpha-secretase, without the cognitive worsening induced by a strong inhibition of the enzyme [37]. Interestingly, a mimic derived from miR29a, named tenomiR, is under evaluation in clinical testing for unrelated pathologies and has been shown to be well tolerated [45].

Two microRNAs—miR-122-5p and miR-451a—regulate Adam10, a key node in the AD network (Table 6 and Table 7), and moderate local connectors in the human interactome (Table 4 and Table 5). Adam10 cleaves App and inhibits the production of amyloid-β (Aβ), which gives it a neuroprotective role in Alzheimer’s disease [46]. We could speculate that inhibiting miR-122-5p and miR-451a with antimiRs may have a beneficial effect on the pathology. However, both miRNAs are under-expressed in Alzheimer’s disease compared to wild-type tissues, suggesting a more complex mechanism of action [47,48].

One reason mimic-based approaches are promising is that microRNAs often regulate multiple genes within a specific pathway or disease module [31]. For example, miR-34a regulates four nodes in the Alzheimer’s disease module presented in Figure 1 and Figure 2 (see also Table 3), and artificial expression of its mimics may therefore have a dramatic effect on the module. Unfortunately, a drug based on a miR-34a mimic named MRX34, which was developed to treat solid tumors, was discontinued as it caused serious immunological adverse reactions [49]. More broadly, mimics-based therapeutics face two major hurdles: they can trigger immune activation and they are difficult to deliver efficiently and safely to the disease-relevant tissue [50]. Still, ongoing advances in delivery systems and molecular design suggest that major technical issues can be overcome in the near future. To date, clinical studies of Alzheimer’s disease have investigated microRNAs exclusively as diagnostic, prognostic or progression biomarkers, while no interventional trials testing microRNA mimics or inhibitors as therapeutic agents have been reported. However, the efficacy of intranasal miR-146a agomiR administration in rescuing cognitive impairment has been demonstrated in mouse models [51].

Observational trials aimed at identifying miRNA signatures associated with AD diagnosis or disease severity from circulating microRNAs retrieved from blood or other fluids have demonstrated the effectiveness of this approach [50,52]. In particular, CognimiR is a panel of 24 microRNAs for early detection of mild cognitive impairment that has shown 90% discrimination accuracy from controls, outperforming other assessments [53]. On the other hand, while last-generation drugs are becoming more appealing, our current understanding of the final effect of microRNA perturbation remains incomplete. The predictive power of the network-based approaches presented in this study can reveal novel information not covered in well-defined pathways and help prioritize microRNA mimics that achieve therapeutic impact with minimal off-target network perturbation, thereby improving the rational selection of candidates for experimental validation and translational development. Our results demonstrate that static centrality metrics and perturbation-based analyses provide complementary information. While degree and betweenness highlight classical hubs, the in silico deletion assay reveals hidden vulnerabilities associated with inter-modular connectors that have disproportionate impact on global cohesion despite modest centrality values. This combined approach provides a more nuanced view of interactome robustness and helps prioritize proteins whose downregulation via mimic-based drugs may have the greatest functional or pathological consequences in Alzheimer’s-related network alterations.

**Figure 5:**
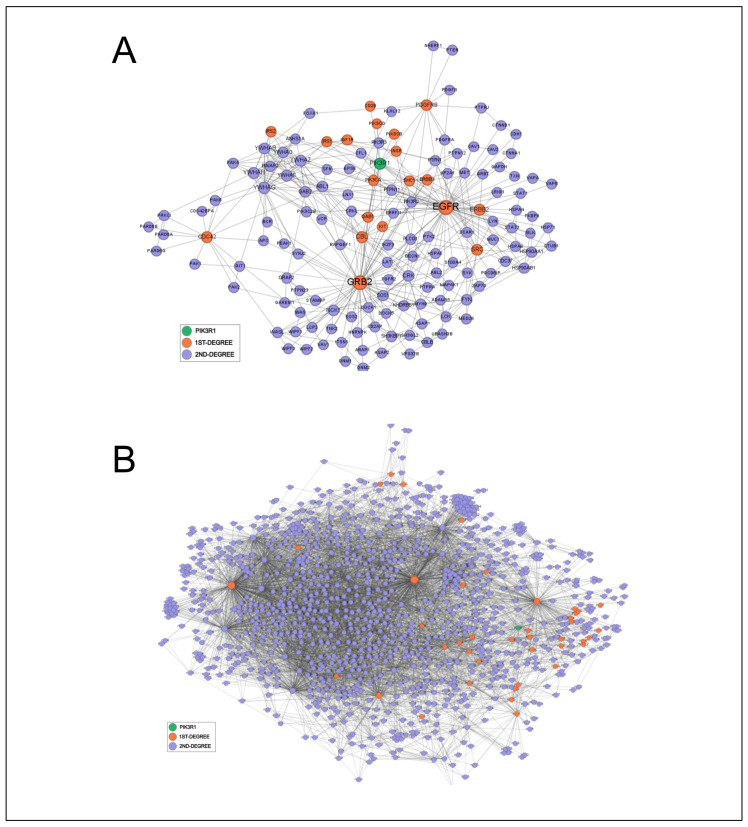
Comparison of a network seeded on PI3KR1 and composed of interactions with a confidence score > 0.65 (**A**) and >0.56 (**B**), showing the relevance of choosing the most appropriate data source in network analysis.

Importantly, the biological significance of these topological dependencies relies heavily on the accuracy of the underlying interaction data. By relying on high-quality interactomes, network analysis gains robustness, reducing the likelihood that structural properties or perturbation effects reflect noise rather than true biological relationships. Figure 5 shows the first and second neighbors of Pi3kr1 in the human interactome (MI score of 0.65 and 0.56), where the second one includes a set of less certain interactions that clearly change its centrality measures. Consequently, accurate biocuration of the literature constitutes a foundational pillar for network medicine studies and for the interpretation of disease-associated perturbations in complex biological systems.

## 5. Conclusions

Artificial intelligence is rapidly transforming network analysis in precision medicine by enabling a far more detailed, mechanistic understanding of how genes, proteins and regulatory elements interact in complex disease states [54,55]. As highlighted recently, AI models and deep-learning systems can now decode subtle regulatory grammar and predict how variations in DNA, chromatin accessibility or transcription-factor binding reshape cellular behavior, insights that are essential for building accurate disease networks [56]. As precision medicine requires predicting how specific interventions will propagate through a patient’s molecular network, AI-driven models offer unprecedented resolution to simulate such perturbations. Network models, however, are very often trained on RNA co-expression data to overcome the limitations of incomplete and noisy information regarding protein–protein and RNA interactions [57]. The availability of interaction networks with the highest possible level of accuracy is crucial, as curated content directly informs hypothesis generation and prediction. A recent work has demonstrated that large language models and agentic systems, trained by expert biocurators, can dramatically accelerate literature curation workflows while maintaining high-quality standards [58], offering a powerful complement to network-science approaches. Models trained with true, verified associations will help identify functional motifs, estimate the impact of mutations, and infer which nodes or edges in regulatory networks are most sensitive to perturbation.

## Figures and Tables

**Table 1:** Interactions retrieved from different databases to build the disease module shown in Figure 1.

Database	Filters	Number of Nodes (N)	Number of Edges (E)
STRING	TaxID: 9606 Type: “Experimental Evidence Only”, “Physical Interactions” Additional Interactors: 10 Confidence Score: ≥0.9	49	56
BioGRID	TaxID: 9606 Type: Interactors w/Physical (LTP) Evidence Cutoff: ≥2 publications (PMIDs) per PPI	117	133
IntAct	TaxID: 9606 Type: “direct interaction” and “physical association” MI Score: ≥0.60	70	74
Final Interactome (duplications deleted)		156	179

**Table 2:** Enrichment of AD-associated genes in the subgraph presented in Figure 1.

Dataset	Number of Genes	Overlap (k)	Background (N)	Odds Ratio	Fisher’s Exact *p*
PPI network	156	26	19,435	14.29	2.2 × 10^−16^
Open Targets	292				

**Table 3:** List of microRNAs regulating nodes in the AD module.

Gene Name	microRNAs
ADAM10	hsa-miR-122-5p; hsa-miR-451a
AKT1	hsa-miR-99a-5p; hsa-miR-542-3p
APOE ^(1)^	hsa-miR-1908-5p; hsa-miR-199a-5p; hsa-miR-650
BACE1	hsa-miR-107; hsa-miR-29c-3p hsa-miR-16-5p
BCL2	miR-125a-5p; miR-140-3p; miR-143-3p; miR-15-5p; miR-16-5p; miR-17-5p miR-181-5p; miR-192-5p; miR-195-5p; miR-204-5p; miR-21-5p; miR-34a-5p miR-429; miR-449a; miR-497-5p; miR-503-5p
CASP3	hsa-miR-138-5p
CDH1	hsa-miR-544a; hsa-miR-9-5p
CTNNB1	hsa-miR-142-3p; hsa-miR-181a-5p hsa-miR-200a-3p; hsa-miR-214-3p135
EP300	hsa-miR-150-5p
GSK3B	hsa-miR-26a-5p; hsa-miR-27a-3p; hsa-miR-99b-3p
HDAC6	hsa-miR-22-3p
MAPK14	hsa-miR-200a-3p
MAPK3	hsa-miR-483-5p
NOTCH1	miR-139-5p; miR-144-3p; miR-30c-5p; miR-34a-5p miR-34b3p; hsa-miR-34c-5p; hsa-miR-449a
PIK3R1	hsa-mir-542-3p
PIN1	hsa-miR-140-5p
PPP1CA	hsa-miR-125b-5p
SGK1	hsa-miR-133b
SIRT1	miR-132-3p; miR-133b; miR-138-5p; miR-155-3p; miR-181a-5p; miR-199a-5p; miR-204-5p; miR-217; miR-22-3p; miR-34a-5p; miR-449a; miR-9-5p
SNCA	hsa-miR-7-5p
SRC	hsa-miR-34a-5p
TGFB1	hsa-miR-211-5p
TGFB2	hsa-miR-7-5p
TRAF6	hsa-miR-146a-5p; hsa-miR-146b-5p
TUBB3	hsa-miR-200c-3p
VDAC1	hsa-miR-320a

^(1)^ The binding of the listed microRNA with the nodes was supported by a “luciferase assay” in at least two different studies, with the sole exception of APOE (one study).

**Table 4:** Centrality measures of nine nodes in the human interactome.

Node Name	Node ID	Degree	Betweenness ^1^	Closeness ^2^
Pik3R1	P27986	19	46,261	0.21
Bace1	P56817	2	1368	0.18
Traf6	Q9Y4K3	26	168,009	0.23
Gsk3b	P49841	19	140,322	0.22
Akt1	P31749	16	107,894	0.22
Cdk2	P24941	26	84,279	0.21
Adam10	O14672	7	30,727	0.18
Mapk3	Q16644	3	4208	0.16
Apoe	P02649	6	100,013	0.17

^1^ Betweenness centrality is given as an absolute value, with higher values indicating that the node appears more often on the shortest paths between pairs of other nodes in the network. ^2^ Closeness centrality was normalized to the maximum value for this network, resulting in values between 0 and 1, where 1 means that the node can reach each of the other nodes in the network in a few steps, while a value closest to 0 means that the node is less likely to influence other nodes.

**Table 5:** Disconnected components after node depletion in the human interactome. The number of components, the largest connected component (LCC) size and the average shortest path length (Avg Path) were computed with the iGraph library from the human network with an MI score of 0.65.

Node Name	Node ID	Components	LCC	Avg Path	Δ Components	Δ LCC	ΔPath Length
No depletion	-	689	7217	5.7726	-	-	-
Pik3r1	P27986	690	7215	5.7733	+1	−2	+0.0007
Bace1	P56817	689	7216	5.7727	0	−1	+0.0001
Traf6	Q9Y4K3	691	7214	5.7774	+2	−3	+0.0048
Gsk3b	P49841	691	7214	5.7760	+2	−3	+0.0034
Akt1	P31749	693	7212	5.7754	+4	−5	+0.0028
Cdk2	P24941	692	7213	5.7737	+3	−4	+0.0011
Adam10	O14672	692	7213	5.7722	+3	−4	−0.0004
Mapk3	Q16644	689	7216	5.7725	0	−1	−0.0001
Apoe	P02649	690	7215	5.7747	+1	−2	+0.0021

**Table 6:** Centrality measures of five nodes in the AD interactome.

Node Name	Node ID	Degree	Betweenness	Closeness
PIK3R1	P27986	1	0	1
Bace1	P56817	2	1.1 × 10^−3^	0.22
Traf6	Q9Y4K3	1	0	0.23
Gsk3b	P49841	4	6.2 × 10^−3^	0.22
Adam10	O14672	6	1.4 × 10^−2^	0.22
Apoe	P02649	3	5.5 × 10^−3^	0.17

**Table 7:** Disconnected components after node depletion in the AD interactome.

Node Name	Node ID	Components	LCC	Avg Path	Δ Components	Δ LCC	ΔPath Length
No depletion	-	100	528	4.7067	-	-	-
PIK3R1	P27986	100	528	4.7067	0	0	+0.0000
Bace1	P56817	100	527	4.7079	0	−1	+0.0012
Traf6	Q9Y4K3	100	527	4.7044	0	−1	−0.0023
Gsk3b	P49841	101	526	4.7079	1	−2	+0.0012
Adam10	O14672	104	519	4.6751	4	−9	−0.0316
Apoe	P02649	101	524	4.669	1	−4	−0.0374

## Data Availability

All data are available in public repositories (IntAct, https://www.ebi.ac.uk/intact/home, accessed on 10 August 2025).

## Supplementary Materials

The following supporting information can be downloaded at https://www.mdpi.com/article/10.3390/biomedicines14010147/s1, Table S1: MicroRNA expression in brain hippocampus and cortex. Values have been normalized in reads per million mapped reads (RPMM). MicroRNAs acting on the considered nodes are highlighted in yellow (high values) or violet (low values); Table S2: Centrality measures in AD interactome MI score > 0.56; Table S3: Node depletion in the AD interactome MI score >0.56; Figure S1. Local network of Apoe showing first- and second-degree interactors extracted from the human IntAct interactome (MI score ≥ 0.65). Nodes are colored as in Figure 4.
