# Supplementary material for: Network Analysis to Identify MicroRNAs Involved in Alzheimer’s Disease and to Improve Drug Prioritization
Source: Biomedicines. 2026 Jan 11;14(1):147. doi: 10.3390/biomedicines14010147 (PMC12839359; doi:10.3390/biomedicines14010147)
Supplement: Supplementary file 1 [file biomedicines-14-00147-s001.zip › Supplementary Table S2.pdf]

Supplementary Table S2 Centrality measures in AD Interactome MI score >0.56

Node name	Node ID	Degree	Betweenness	Closeness
PIK3R1	P27986	42	$5.29e^{-3}$	0.38
Bace1	P56817	4	$1.3\ e^{-4}$	0.3
Traf6	Q9Y4K3	3	$2.93e^{-6}$	0.26
Gskbeta	P49841	8	$1\ e^{-3}$	0.32
Akt1	P31749	14	$6.6\ e^{-4}$	0.35
Cdk2	P24941	4	$2.06e^{-6}$	0.26
Adam10	O14672	7	$3.2\ e^{-3}$	0.31
