# Supplementary material for: Network Analysis to Identify MicroRNAs Involved in Alzheimer’s Disease and to Improve Drug Prioritization
Source: Biomedicines. 2026 Jan 11;14(1):147. doi: 10.3390/biomedicines14010147 (PMC12839359; doi:10.3390/biomedicines14010147)
Supplement: Supplementary file 1 [file biomedicines-14-00147-s001.zip › Supplementary Table S3 .pdf]

Supplementary Table S3 Node depletion in the AD Interactome MI score >0.56

Node name	Node ID	Components	LCC size	Avg Path	Δ Comp.	Δ LCC Size	ΔPath Length
No depletion	-	39	3464	1.921667	-	-	-
PIK3R1	P27986	41	3461	1.922658	+2	-3	+0.000991
Bace1	P56817	39	3463	1.921619	0	–1	-0.000048
Traf6	Q9Y4K3	39	3463	1.921523	0	–1	-0.000144
Gskbeta	P49841	40	3462	1.921553	+1	–2	+0.000114
Akt1	P31749	39	3463	1.921982	0	–1	+0.000315
Cdk2	P24941	39	3463	1.921555	0	–1	+0.000112
Adam10	O14672	41	3461	1.921199	+3	–4	–0.000548
