# Supplementary figures and images for: Network Analysis to Identify MicroRNAs Involved in Alzheimer’s Disease and to Improve Drug Prioritization
Source: Biomedicines. 2026 Jan 11;14(1):147. doi: 10.3390/biomedicines14010147 (PMC12839359; doi:10.3390/biomedicines14010147)

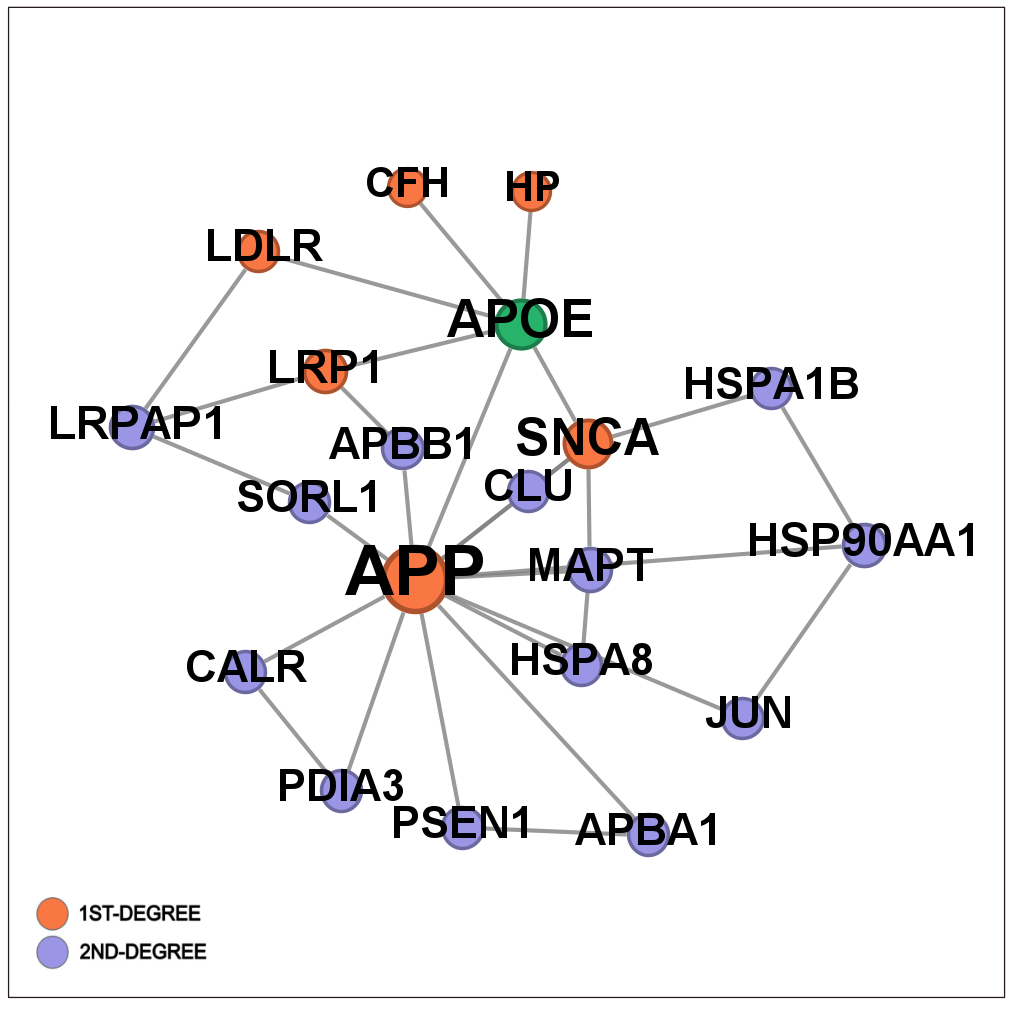

Supplement: Supplementary file 1 [file biomedicines-14-00147-s001.zip › Suppl Figure S1.jpg]
